# Tripartite ATP-Independent Periplasmic (TRAP) Transporters and Tripartite Tricarboxylate Transporters (TTT): From Uptake to Pathogenicity

**DOI:** 10.3389/fcimb.2018.00033

**Published:** 2018-02-12

**Authors:** Leonardo T. Rosa, Matheus E. Bianconi, Gavin H. Thomas, David J. Kelly

**Affiliations:** ^1^Department of Molecular Biology and Biotechnology, University of Sheffield, Sheffield, United Kingdom; ^2^Department of Animal and Plant Sciences, University of Sheffield, Sheffield, United Kingdom; ^3^Department of Biology, University of York, York, United Kingdom

**Keywords:** solute transport, periplasmic binding-proteins, secondary transporter, high-affinity, carboxylic acids

## Abstract

The ability to efficiently scavenge nutrients in the host is essential for the viability of any pathogen. All catabolic pathways must begin with the transport of substrate from the environment through the cytoplasmic membrane, a role executed by membrane transporters. Although several classes of cytoplasmic membrane transporters are described, high-affinity uptake of substrates occurs through Solute Binding-Protein (SBP) dependent systems. Three families of SBP dependant transporters are known; the primary ATP-binding cassette (ABC) transporters, and the secondary Tripartite ATP-independent periplasmic (TRAP) transporters and Tripartite Tricarboxylate Transporters (TTT). Far less well understood than the ABC family, the TRAP transporters are found to be abundant among bacteria from marine environments, and the TTT transporters are the most abundant family of proteins in many species of β-proteobacteria. In this review, recent knowledge about these families is covered, with emphasis on their physiological and structural mechanisms, relating to several examples of relevant uptake systems in pathogenicity and colonization, using the SiaPQM sialic acid uptake system from *Haemophilus influenzae* and the TctCBA citrate uptake system of *Salmonella typhimurium* as the prototypes for the TRAP and TTT transporters, respectively. High-throughput analysis of SBPs has recently expanded considerably the range of putative substrates known for TRAP transporters, while the repertoire for the TTT family has yet to be fully explored but both types of systems most commonly transport carboxylates. Specialized spectroscopic techniques and site-directed mutagenesis have enriched our knowledge of the way TRAP binding proteins capture their substrate, while structural comparisons show conserved regions for substrate coordination in both families. Genomic and protein sequence analyses show TTT SBP genes are strikingly overrepresented in some bacteria, especially in the β-proteobacteria and some α-proteobacteria. The reasons for this are not clear but might be related to a role for these proteins in signaling rather than transport.

## Solute Binding-Protein (SBP) dependant secondary transporters: the TRAP and TTT systems

**Figure 1:**
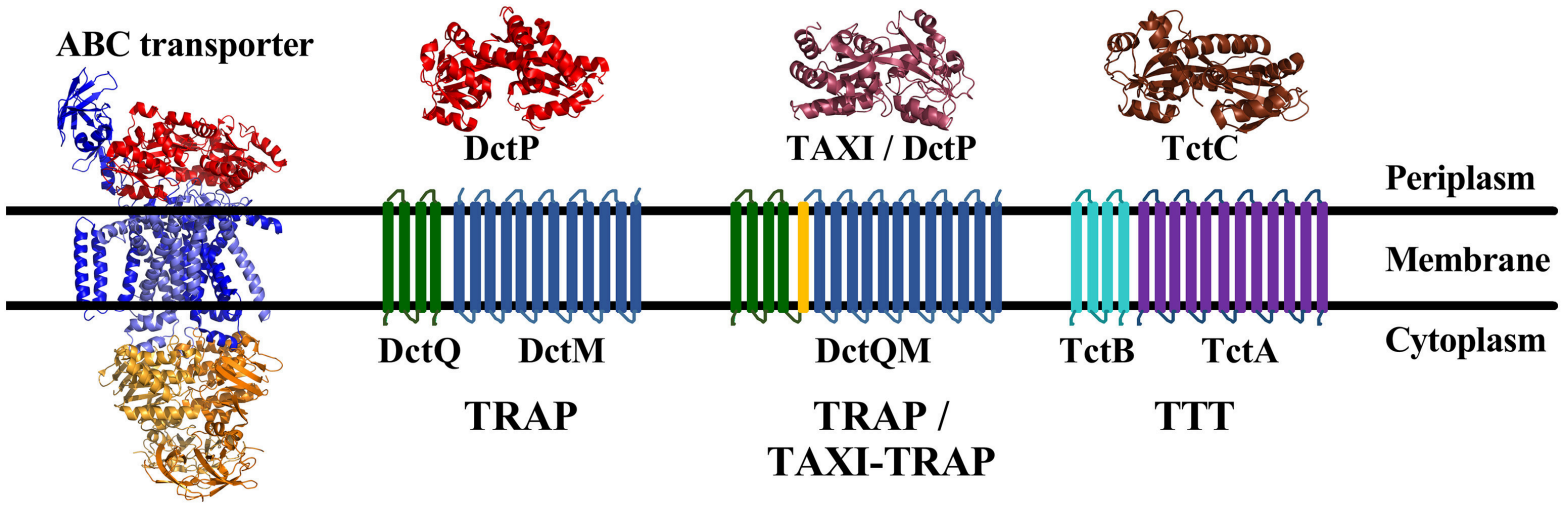
Overall topologies and structures of the different Solute Binding Protein (SBP dependant) transporter families. The ABC transporters are represented by the *E. coli* maltose transporter MalEFGK2 (PDB 2R6G). It is composed of a solute binding protein (red), two Transmembrane (TM) domains (blue), and two Nucleotide binding domains (NBD) (orange); The secondary Tripartite ATP-independent periplasmic (TRAP) Transporters are composed of a 12 TM domain channel DctM (blue) and a 4 TM domain protein DctQ (green), which can be fused together by an additional TM domain (yellow) in a DctQM protein, and a DctP or TAXI SBP protein, represented respectively by the SiaP from *H. influenza* (PDB 2CEY) (light red) and the TT1099 from *T. thermophilus* (PDB 1US4) (dark red). TAXI-TRAP were always found associated with fused DctQM proteins; The Tripartite Tricarboxylate Transporter (TTT), is formed also by a 12 TM channel TctA (purple), a 4 TM protein TctB (cyan) and a TctC solute binding protein, represented by Bug27 from *B. pertussis* (PDB 2QPQ) (Brown). In some rare cases, TctAB proteins may be also fused. Although sharing similar topology, the TRAP and TTT systems share no sequence similarity.

Solute Binding-Protein (SBP) dependent transport systems contain, in addition to the membrane proteins, a soluble extra-cytoplasmic protein, located either free in the periplasm or anchored to the membrane in the case of Gram-positive bacteria, which binds the substrate with high affinity, and specificity, allowing uptake even in very low concentrations of ligands. Three families of SBP dependant transporters are currently known, the composition of which are summarized in Figure 1. ATP-binding cassette (ABC) transporters use the free energy of ATP binding and hydrolysis to move substrates across the membrane against a concentration gradient. First described in the early 1970‘s (Kalckar, 1971; Willis and Furlong, 1974), this family is by far the best investigated SBP-dependant transporter family, with the maltose and vitamin B_12_ uptake systems as the most thoroughly studied models, and was subject of several reviews over recent years (Jones and George, 2004; Davidson et al., 2008; Rice et al., 2014; Maqbool et al., 2015; Wilkens, 2015; Locher, 2016).

The Tripartite ATP-independent periplasmic (TRAP) transporters (TC: 2.A.56) and Tripartite Tricarboxylate Transporters (TTT, TC: 2.A.80), on the other hand, use ion-electrochemical gradients to move substrates in a symporter mechanism, thus being defined as secondary transporters. These two families are significantly less well-understood than ABC systems but share a similar overall protein composition and topology, as well as genomic organization. In addition to the SBP‘s (“P” subunit in TRAP systems, “C” subunit in TTT), each system is comprised of two transmembrane proteins, one well-conserved 12 transmembrane (TM) domain protein (“M” subunit in the TRAP systems, “A” subunit in TTT) and one poorly conserved 4 TM domain protein (“Q” subunit in TRAP systems, “B” subunit in the TTT) (Forward et al., 1997; Winnen et al., 2003; Thomas et al., 2006; Hosaka et al., 2013, Figure 1). However, no sequence similarity is found between the corresponding proteins in these families, thus representing either a case of convergent evolution (Fischer et al., 2010) or very ancient orthology and divergence (Winnen et al., 2003).

Regardless of their lack of sequence similarity, the SBPs from these two families show very similar tertiary structures. They are folded in a “Venus fly-trap” shape, with two wings composed of one β-sheet containing four to six strands, surrounded by α-helices and connected by a hinge. Opened in the *apo* form, the wings close around the substrate in a very specific manner, binding the substrate tightly in a cleft formed between the two domains. The enclosure of the substrate then allows the protein to interact with the transmembrane domains (Herrou et al., 2007). It is suggested that these two wings were generated by a duplication event in early TTT (and other SBP dependent) transporters (Winnen et al., 2003). Classification of SBPs into related clusters has been proposed, based on their secondary and tertiary structural patterns and their substrate specificities, with the first classification into three distinct types proposed by Fukami-Kobayashi et al. (1999). With the exponential increase in new entries for SBPs in genomic databases due to new sequencing capabilities, it became clear that the separation into three types was too simplistic to comprise SBP diversity, and thus a new model was presented by Berntsson et al. (2010) and recently revised by Scheepers et al. (2016). Both TRAP and TTT SBP‘s are contained within the Type II group in the first classification, and inside Cluster E in the latter. This review summarizes the evidence of a relationship between these two classes of secondary high-affinity uptake systems and pathogenicity. Additionally, it adds an evolutionary perspective regarding the expansion of the TTT family in some pathogens.

## The TRAP transporter family

The first characterization and naming of TRAP transporters was described in *Rhodobacter capsulatus* by Forward et al. (1997), when a SBP encoding gene was found adjacent to two genes encoding transmembrane proteins of 12 (DctM) and 4 (DctQ) predicted helices. Functional studies showed symport of C4-dicarboxylic acids apparently energized by the proton motive-force. Subsequent studies showed that these systems can transport a variety of substrates under different contexts. Detailed reviews about this family were provided by Kelly and Thomas (2001) and Mulligan et al. (2011), and the following sections will focus on more recent insights.

### Substrate diversity of the TRAP family and roles in pathogenicity

**Figure 2:**
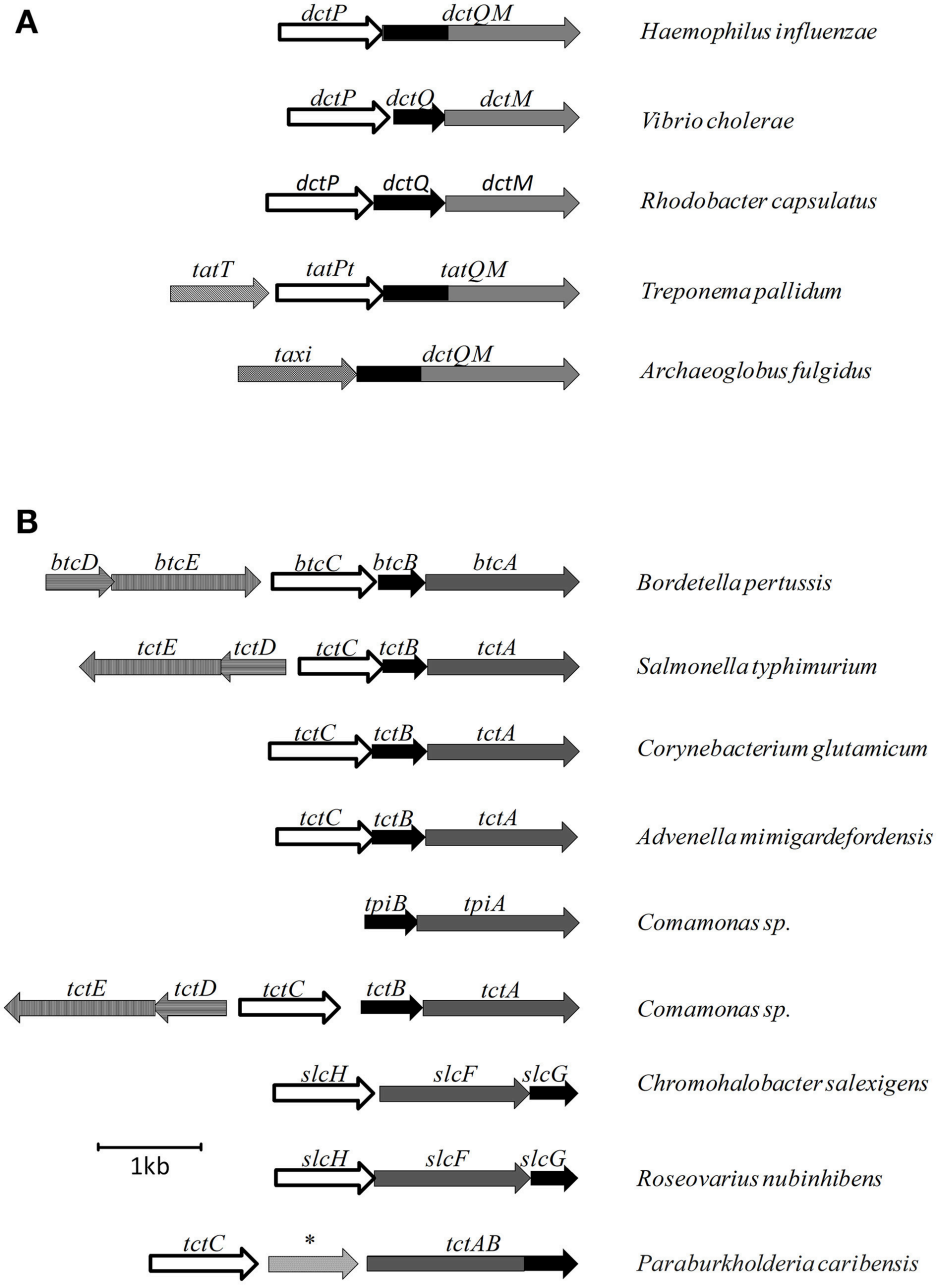
Examples of genetic organization for different secondary SBP dependant transporters. **(A)** Gene organizations for TRAP systems. **(B)** Gene organization for TTT systems. ^*^ represents an amido-hydrolase gene.

The best studied TRAP system is undoubtedly SiaPQM from *Haemophilus influenzae* (Figures 1, 2A), discovered by Severi et al. (2005) to be involved in the uptake of sialic acid. Sialic acid is a generic name for a class of 9-carbon sugar acids used by most eukaryotic cells in the form of cell surface glycoproteins. For this reason, many pathogens evolved to mimic these surface structures in their own cell envelope, constituting an important virulence factor which improves evasion of the human immune system (Bouchet et al., 2003). In *H. influenzae*, absence of SiaPQM causes loss of sialic acid uptake and lack of incorporation in the lipo-oligossacharide (Allen et al., 2005), and a subsequent study showed an increased susceptibility of this pathogen to human serum and decreased virulence in the chinchilla otitis model (Jenkins et al., 2010). Systems homologous to SiaPQM were subsequently found to be involved in the uptake of sialic acid in several pathogens, such as *Vibrio cholerae, Fusobacterium nucleatum*, and *Vibrio vulnificus* (Severi et al., 2005), the latter being shown to transport sialic acid in 67 clinical isolates (Lubin et al., 2012). Signature-tagged mutagenesis studies in *Pasteurella multocida*, an opportunistic pathogen of livestock, showed that disruption of genes related to sialic acid metabolism resulted in decrease of virulence in mice models (Fuller et al., 2000). A subsequent study showed that a SiaP homolog was involved in the uptake of sialic acid in this bacterium (Steenbergen et al., 2005), and that disruption of sialic acid uptake resulted in decreased virulence in a turkey model (Tatum et al., 2009). Severi et al. (2007) provides a review of how sialic acid uptake and metabolism is used as a virulence factor in different pathogens, and Vimr et al. (2004) provides a more general review about sialic acid metabolism. Thomas (2016) gives a recent overview of the different uptake strategies and transport systems used by different pathogens for the uptake of sialic acid.

In *Bordetella pertussis*, the causative agent of whooping cough, two DctP homologs are encoded in the vicinity of virulence-related operons modulated by the BvgA/BvgS two-component system. The two proteins were crystalized by Rucktooa et al. (2007) with a pyroglutamic acid bound in the substrate cleft. One of these proteins is highly expressed in *B. pertussis*, although the membrane components of the system seem to be mutated and non-functional. Although it is unclear what physiological role pyroglutamic acid would have, this amino-acid is present in the filamentous hemagglutinin produced by *B. pertussis*, and it was speculated it could serve as a glutamate reserve. In fact, BugE, an abundantly expressed SBP from the TTT family also was shown to bind glutamate (Huvent et al., 2006b), suggesting glutamate metabolism might play an important role in the pathophysiology of this bacterium.

TRAP systems are very important also in environmental organisms and in biotechnologically relevant processes. In *Halomonas elongata*, Grammann et al. (2002) showed that the TeaABC operon was responsible for accumulation of the compatible solute ectoine, in response to osmolarity stress, and that this transporter was osmoregulated. In *R. capsulatus* a TRAP system was shown to be involved in the import of several monocarboxylic 2-oxo-acids involved in amino-acid biosynthesis (Thomas et al., 2006). Chae and Zylstra (2006) showed the involvement of TRAP transporters in the degradation of several benzoate derivatives, including toxic chlorinated aromatics. In *Rhodopseudomonas palustris* the TarPQM system was shown to be involved in the degradation of lignin-derived aromatic compounds, in a redundant function also executed by an ABC transporter in the same gene cluster (Salmon et al., 2013). GaaPQM from *Agrobacterium tumefaciens* was described to be involved in plant virulence (Zhao and Binns, 2016). Maimanakos et al. (2016) showed that TRAP transporters are found in the vicinity of arylmalonate decarboxylases (AMDases) and recently, Meinert et al. (2017) showed a TRAP system involved with the uptake of five different sugars in *Advenella mimigardefordensis*, but only after they have been converted to their respective sugar acids in the periplasm (Thomas, 2017).

Vetting et al. (2015) published a highly significant study, which multiplied several times our understanding about substrate specificity in TRAP systems. 8,240 SBP‘s were used to build a sequence similarity network, grouping them into several clusters. From these, 304 representatives of non-characterized groups were then screened, coupling differential scanning fluorescence, crystallography and mass spectrometry of co-purified ligands. The methodology shows the importance of using complementary methods and proposes an efficient strategy for the study of SBP‘s. As a result, 71 of the isofunctional clusters had a ligand assigned; 69 high-resolution crystal structures were obtained; previously known ligands were assigned to non-characterized clusters and several new ligands were found to be captured by TRAP transporters, such as D-glucuronate/D-galacturonate, 6-carbon aldonic acids, cell-wall constituents, lipopolysaccharide components, glycerol-3-phosphate/diglycerol-phosphate, 2-acetolactate, orotic acid, indole acids, pantoate/D-erythonate, and ethanolamine, this last being a particular surprise due to its positive charge in contrast to the typical negatively charged carboxylates of most other TRAP transporter substrates. This work was done as part of the Enzyme Function Initiative (EFI), a network aiming to characterize the biochemical and physiological function of different classes of enzymes, among which are soluble binding proteins, through high-throughput sequence/structure based strategies (http://www.enzymefunction.org/).

### The neglected group: TAXI-TRAP transporters

It was observed by Kelly and Thomas (2001) that, in some cases, the SBPs associated with the DctQM subunits in the genome showed very limited sequence similarity to DctP, forming a distinct group, TRAP associated extracytoplasmic immunogenic (TAXI) proteins, named after an immunogenic protein of unknown function from the pathogen *Brucella* (Mayfield et al., 1988). A previous study by Rabus et al. (1999) had found some similarity between TAXI proteins and the *E. coli* glutamate binding protein, and the only structure available for a TAXI protein, generated by Takahashi et al. (2004), reinforced these initial findings, as it was described as a glutamate/glutamine binding protein. However, the deletion of a TAXI protein from *Psychrobacter arcticus* was shown by Bakermans et al. (2009) to affect growth also in other dicarboxylic acids such as acetate, butyrate and fumarate. TAXI-TRAP systems usually have the DctQM subunits fused (Figures 1, 2A) and, because they are found also in many Archaea species, it is believed that this system is an ancient form of TRAP transporter. Although Mulligan et al. (2011) provided a brief speculation about potential function of TAXI-TRAP systems based on their genomic context, a complete characterization of this group is still to be generated.

### Two is not enough: the TPAT system

In addition to the classical TRAP and the TAXI-TRAP transporters, a third class of TRAP system was characterized by Deka et al. (2012) in the pathogen *Treponema pallidum*. *T. pallidum* is the causative agent of syphilis, a disease which continues to be a challenge in global health. This organism is an obligatory pathogen, which lacks many vital biosynthetic pathways for nucleotides, lipids, and most amino-acids, relying on transport systems to obtain these vital requirements from the human host (Radolf et al., 2016). Deka et al. (2012) observed the existence of a single operon encoding a TRAP system in *T. pallidum* genome, composed of three genes, as shown in Figure 2A. One *dctP* and one *dctQM* homolog, named *tatP*_*T*_ and *tatQM*, and a third gene of unknown function, named *tatT*. The biochemical and crystallographic characterization of TatT showed this soluble protein was formed by 13 α-helixes and one small helix, structured around a central hydrophobic pore which opened to both ends of the structure. Some of these helices were homologous to a tetratricopeptide motif (TPR), normally involved in protein-protein interactions (D'Andrea and Regan, 2003), which gave the name for this group of TRAP transport systems as TPR-protein associated transporters (TPAT). Using cross-linking, western blotting, analytical ultracentrifugation and computational modeling, it was shown that TatT formed a trimer, which in turn interacted with three subunits of the DctP homolog TatP_T_ (Deka et al., 2012). A later study by Brautigam et al. (2012) confirmed these predictions through crystallization of the TatT and TatP_T_ complex. In these structures, it was shown that the substrate cleft from TatP_T_ was aligned to the C-terminal side of the pore in TatT, with minor structural changes happening upon complexation, the main one being the displacement of one loop from TatP_T_ domain 2 in contact with the binding cleft, called a “cleft-finger.” The hydrophobicity observed both in TatT pore and TatP_T_ cleft, together with the presence of a linear hydrophobic molecule crystalized in the TatT pore, suggested that this system is involved in the uptake of hydrophobic molecules (Deka et al., 2012). As both proteins are found *in vivo* as lipoproteins, anchored to the membrane, it was suggested as a mechanism that this interaction created a chaperone environment for the transport of lipids through the periplasmic hydrophilic environment, where TatT would receive the lipid from the host, anchored in the outer membrane, and transfer it to TatP_T_, anchored in the inner membrane, which in turn would deliver it to the TatQM subunit (Brautigam et al., 2012). TPAT systems were found in 35 other species, among other spirochaetes and also among free-living proteobacteria. In this latter group, it was mostly found in species capable of degrading hydrocarbons, reinforcing the potential role in aliphatic transport this distinct group of TRAP transporters might have.

### Biochemical and functional studies of the DctQM subunits

Unlike the ABC transporters, no crystal structures have been obtained to date regarding the membrane components of TRAP systems, however some mechanistic information is available particularly regarding energy-coupling. In many systems, such as SiaPQM from *H. influenzae*, the DctM and DctQ membrane units are not expressed separately, but fused in one only protein containing 17 transmembrane helices, one more than expected due to an additional helix that connects the cytoplasmic C-terminal part of DctQ with the periplasmic N-terminal part of DctM (Figure 2A, Mulligan et al., 2009). Even when expressed separately, DctM and DctQ were shown to form a tight complex with a 1:1 stoichiometry during the folding procedure, and attempts to separate the two proteins resulted in disruption of function (Mulligan et al., 2012). While DctM is believed to form a translocation channel and is a member of the ion transporter superfamily (Rabus et al., 1999), the role of DctQ has not been established yet and has a much more variable sequence. It is known that it is essential for transporter function and it was suggested that DctQ might act to mediate interactions between DctM and DctP, chaperoning DctM and stabilizing it in the membrane or participating in energy coupling (Wyborn et al., 2001). Mulligan et al. (2009) performed a series of experiments showing that the presence of Na^+^ ions was required for sialic acid transport via SiaPQM in *H. influenzae*. Replacement of Na^+^ for Li^+^ ions did not result in uptake activity, and although neither ΔpH or Δψ alone resulted in transport in absence of Na^+^, the gradients were able to promote substrate uptake when Na^+^ was present in equal concentrations in both sides of the membrane. These results show that substrate uptake in TRAP transporters is Na^+^ dependent and characterized as an eletrogenic process, where at least two Na^+^ ions are co-transported. Not surprisingly, the TRAP family is widely found in bacteria living in saline environments, using the naturally provided Na^+^ gradient to provide substrate uptake, as discussed by Mulligan et al. (2007). In addition, Mulligan et al. (2009) showed that in opposition to conventional secondary transporters such as the ones from the MFS family, the transport in the TRAP family is unidirectional. The substrate transporter exposes the binding cavities alternatively in the cytoplasm and the periplasm, but because in TRAP transporters the exposure in the periplasmic side only occurs when in interaction with the SBP, movement in the opposite direction is blocked, even when gradients are inverted. The only condition in which contrary movement was observed was in the presence of an excess of un-liganded SiaP in the periplasm, but these conditions are not physiologically relevant. In addition, it was shown that replacement of the SiaP in *H. influenzae* (HiSiaP) by an homolog from *V. cholerae* (VcSiaP) did not complement its function, suggesting that the interactions between DctP proteins and the membrane counterparts are specific in each case, rather than promiscuous among the family (Mulligan et al., 2009). Mulligan et al. (2012) performed these same transport assays and characterization of the SiaQM subunits in the homologous system from *V. cholerae*, which comprises a true tripartite system instead of the fused subunits. The results from this study were very similar to the *H. influenzae* fused SiaQM system.

### Crystal structure and dynamics of TRAP SBP's

**Figure 3:**
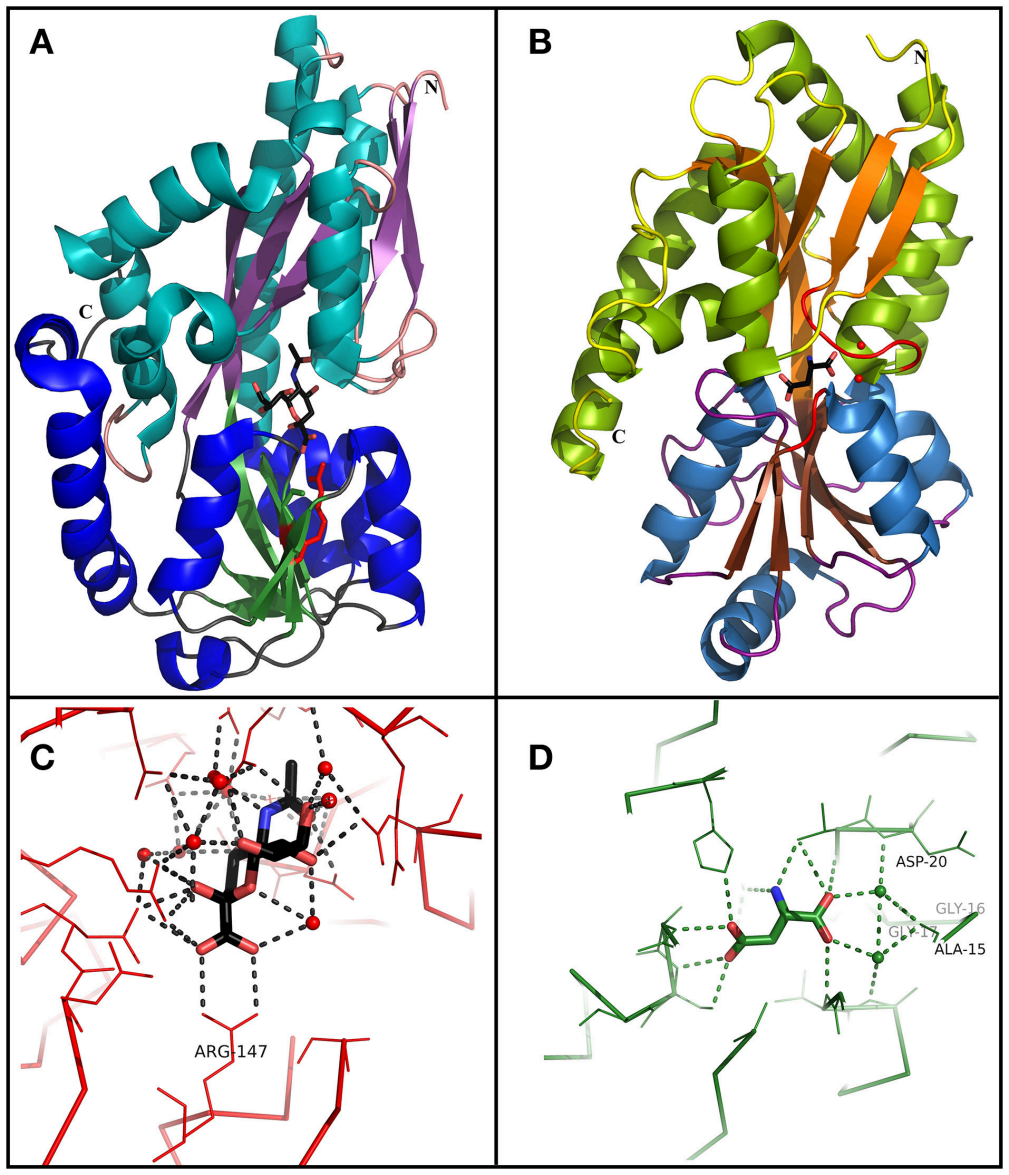
Comparison between TRAP and TTT SBP crystal structures. **(A)** Overall structure of SiaP, a sialic acid binding SBP from the TRAP family in *H. influenzae* (PDB 3B50). Domain 1 is represented in cyan (α-helix) and purple (β-sheet), and domain 2 is represented in blue (α-helix) and green (β-sheet). A sialic acid molecule is shown in the binding pocket. Arg147, important to perform a salt bridge with the carboxylic group of the substrate in most SiaP homologs is shown in red. **(B)** Overall structure of BugD, a aspartate binding SBP from the TTT family in *B. pertussis* (PDB 2F5X). Domain 1 is represented in green (α-helix) and orange (β-sheet), and domain 2 is represented in blue (α-helix) and brown (β-sheet). An aspartate molecule is shown in the binding pocket. Two loops, between β1 and α1 and between β7 and α6, are involved in the conserved coordination of two water molecules, which bridge hydrogen bonds with the proximal carboxylic group in the substrate. These loops and waters are shown in red **(C)** Binding pocket of SiaP, showing the coordination of the sialic acid molecule. Arg147 perform a salt bridge with the carboxylic group in the substrate. The remaining of the molecule is coordinated by hydrogen bonds and water molecules which are variable inside the TRAP family. **(D)** Binding pocket of BugD, showing the coordination of the aspartate molecule. The residues Ala-15, gly-16, gly-17, and Asp-20 participate in the loop between β1 and α1, coordinating two water molecules which bridge hydrogen bonds with the proximal carboxylic group in the substrate. This coordination is very conserved among the TTT family. The remaining substrate coordination occur through non-conserved hydrogen bonds and water bridging.

The first crystal structure of a TRAP SBP was the SiaP protein from *H. influenzae* (Müller et al., 2006). TRAP SBP‘s have wings very similar to the Type II proposed structure by Fukami-Kobayashi et al. (1999), but with a remarkably large single β-strand, which connects both domains and participates in both β-sheet domains (Figure 3A). In addition, this family contains a long α-helix, which spans both domains and kinks upon ligand binding. These features characterize the TRAP transporters in Cluster E of the division proposed by Scheepers et al. (2016). The hinge-bending upon ligand-binding was estimated by Müller et al. (2006) to be ~30Å based on comparison between unliganded and ligand protein crystals.

Although ligand positioning inside the binding pocket is conserved, the hydrogen bonds and hydrophobic interactions for each molecule coordination vary, making substrate prediction difficult for this family. A conserved arginine residue in domain 2, however, turns out to be crucial for ligand interaction (Figure 3C), as discussed by Fischer et al. (2010, 2015). Localized in β-strand 6, which is in a stable β-sheet, the side chain of this highly conserved residue (96.8% of 6,142 sequences searched) points toward the binding cavity and, unusually, is stabilized through a hydrophobic patch and a hydrogen bond, with a bending in Cβ which allows the side chain to reach the pocket (Figures 3A,C). In the presence of ligand, it makes a salt bridge with the ligand carboxylate group, believed to be the first step in ligand coordination. This interaction is believed to be critical for proper functioning of most SBPs from the TRAP family as high-affinity binding proteins, although it is not essential for the coordination of domain closure upon ligand binding (Fischer et al., 2015). The TatP_T_ homologs, believed to be involved in the uptake of aliphatic substrates, mostly lack this residue, having, instead, an alanine (Deka et al., 2012). Mutations of this arginine residue in SiaP were shown by Johnston et al. (2008) to disrupt sialic acid uptake in *H. influenzae* and recently Fischer et al. (2015) showed that replacing it by a lysine decreased the binding affinity for sialic acid by SiaP from 0.14 to 38.7 μM, and mutating it to an alanine resulted in no binding. Crystallization of these two mutant proteins, however, showed minor differences in ligand coordination, where in place of the missing N atoms, coordinated water molecules bridged the carboxylic group of the ligand to the protein, dissipating the negative charge. Subsequent growth experiments showed that cell growth could be restored in the presence of high external concentrations of sialic acid, as the higher concentration would compensate for the weaker affinity; In the same study, it is shown also that this water coordination enables a higher promiscuity in the binding pocket, allowing it to coordinate an analog ligand containing an amide group in place of the carboxylic acid. In addition, PELDOR spectroscopy analysis recently performed by Glaenzer et al. (2017) showed that no intermediate state of VcSiaP is observed in solution upon ligand binding, which can only be in an open or closed conformation. Moreover, the protein does not alternate to closed conformation unless the ligand is present, a fact that supports the current model in which the SBP will only return to the open conformation upon interaction with the membrane components, avoiding unproductive opening and closing of the binding protein (Mulligan et al., 2011).

In addition, the variable positioning of helix 3 across different proteins seem to be responsible for the adaptation of the binding pocket for different ligand sizes, given by structural changes in regions flanking this helix (Lecher et al., 2009). In some cases, generally for smaller TRAP ligands, cation atoms are also required for ligand coordination. However, as shown by Akiyama et al. (2009), these cations are usually non-specific, and have a structural role to bridge the interaction with the protein chain, and are not necessary when the ligands are capable of filling the respective space and interact directly (Fischer et al., 2010).

Although most TRAP SBPs are found to act as monomers, there is evidence that some of them might require dimerization for function. Gonin et al. (2007) showed that TakP, a pyruvate binding protein from *Rhodobacter sphaeroides*, crystalized as a dimer, and the functional importance of dimerization was validated by tryptophan fluorescence quenching, gel filtration and cross-linking experiments. The dimerization is believed to occur through a kinked C-terminal helix, which swaps its position with the same portion of the dimer counterpart. Additionally, Akiyama et al. (2009) crystalized a lactate binding protein from *Thermus thermophilus* which interacts back-to-back in a dimerization process stabilized by hydrophobic interactions in the C-terminal region of the protein. Finally, Cuneo et al. (2008) confirmed also the dimerization state of a TRAP protein from *Thermotoga maritima* through gel filtration analysis and X-ray scattering. It remains unclear, however, how this dimerization process would promote or interfere with the transport mechanism when interacting with the DctQM subunits.

## The TTT family

As with the TRAP transporters, systems in the TTT family are composed of a conserved 12 TM protein, (TctA homologs) believed to act as a symport protein energized by an electrochemical ion-gradient (although this has not been experimentally determined) and poorly-conserved 4 TM protein (TctB homologs) with unknown function, in combination with an SBP (TctC homologs) which binds the substrate with high affinity (Figure 1, Sweet et al., 1979; Winnen et al., 2003). However, the TTT family has not been subject to many experimental studies and knowledge about this family is still scarce; the topic being last reviewed by Winnen et al. (2003).

### Substrate diversity of the TTT family: role of the solute binding-protein and occurrence in pathogens

As the prototype for the TTT family, the TctC citrate transporter was first described by Sweet et al. (1979) in the pathogen *Salmonella typhimurium*, one of the most important causative agents of food-borne gastrointestinal infections and a growing problem due to the recent emergence of multidrug resistant strains (Hur et al., 2012). TctC was found to be involved in the uptake of the tricarboxylic acid citrate with low-μM affinity, with citrate uptake severely reduced in this organism upon disruption of the *tctC* gene. This function gave the name to the family as tricarboxylate transporters (Sweet et al., 1979; Somers et al., 1981). A series of 36 tricarboxylate and di-carboxylate metabolites were later shown by Sweet et al. (1984) to inhibit citrate binding to TctC to varying extents, suggesting that the substrate range for this protein might not be restricted to citrate. Genetic mapping studies initiated by Somers and Kay (1983) and finished by Widenhorn et al. (1988) showed that downstream of the *tctC* locus there were two more encoded proteins, of 19 and 45 kDa, corresponding, respectively, to the transmembrane proteins TctB and TctA (Figures 1, 2B). The gene arrangement of *tctCBA*, is similar to that found for the majority of TRAP transporters (Mulligan et al., 2011). Encoded in the opposite direction, a fourth gene, *tctD*, was shown by Widenhorn et al. (1989) to encode a transcription regulator of the *tctCBA* operon, which was found to be repressed when *tctD* was deleted or in the presence of glucose in the medium. Homologous systems to TctCBA are found in many bacteria, mainly Proteobacteria, and citrate uptake is the commonest identified role for the few other TTT systems experimentally characterized to date (Antoine et al., 2003; Brocker et al., 2009; Hosaka et al., 2013; Graf et al., 2016). Citrate has been shown to act in some cases as an iron chelator for different transport systems (Yancey and Finkelstein, 1981; Braun, 2001; Luck et al., 2001; Banerjee et al., 2016), and although the potential role of TctC acting as an iron transport protein has not been investigated to date, experiments performed with *S. typhimurium* (Sweet et al., 1979) showed that citrate binding to TctC is improved in the presence of Na^+^ Ca^2+^, Mn^2+^ and Fe^2+^, while partially inhibited by Mg^2+^, Ni^2+^, Zn^2+^ and Co^2+^. In addition, growth experiments performed by Brocker et al. (2009) using a homologous TctCBA system from *Corynebacterium glutamicum* showed that this system was able to uptake citrate in the presence of Ca^2+^ and Mg^2+^, but not Sr^2+^.

After the characterization of TctC, all proteins homologous to the TctCBA systems in newly released genomes were annotated either as unknown proteins or citrate uptake systems, and this family was neglected for over a decade, until one TctC homolog was found by Antoine et al. (2000) to be encoded upstream of the pertussis toxin (PTX) virulence island, one of the most important toxins produced by the causative agent of whooping cough, *B. pertussis*. This gene was found to be conserved in this locus for different *Bordetella* species and was named *bugT*, standing for “*Bordetella* uptake gene.” Although a relationship between the BugT protein and the production of PTX was not confirmed, regions coding for other virulence factors, such as the adenylate cyclase toxin (AC) and the dermonecrotic toxin (DNT) also contained *bugT* homologs, while two *bug* homologs were negatively regulated by the BvgAS two-component system, responsible for the activation of virulence factor production (Antoine et al., 2000, 2003). Recently, a single-nucleotide-polymorphism (SNP) in one *bug* gene was consistently identified in an Australian epidemic strain of *B. pertussis* (Safarchi et al., 2016). As discussed in the next sections, further searches in the *B. pertussis* genome found homologs of Bug proteins to be extensively overrepresented, with 76 genes encoding distinct homologs (Antoine et al., 2000). In contrast, only two sets of genes coding for transmembrane proteins homologous to *tctAB* were found in *B. pertussis*, and most of the BugT homologs showed no obvious membrane counterparts encoded in their genomic vicinity, hence the designation of them as “orphan” proteins.

The only complete operonic encoded TTT system in *B. pertussis*, encoded by *bctCBA*, contains *bug4* as the *tctC* homolog, and was found to be the equivalent of *tctCBA* from *S*. *typhimurium*, as expression was upregulated by citrate and gene disruption resulted in lower citrate uptake rates (Antoine et al., 2003). As shown by Antoine et al. (2005), upstream of the *bctCBA* operon is encoded the two-component system *bctDE*, transcribed in the same direction but forming a separate operon (Figure 2B), which showed a basal expression level independent of citrate. When this two-component system was deleted, expression of *bctCBA* was not detected, showing that *btcDE* was in fact activating transporter gene expression. Disruption of the *bctBA* components, on the other hand, increased operon expression, due to an accumulation of citrate in the periplasm to be directed to signaling purposes (Antoine et al., 2005). Finally, when *bctC* was deleted, *bctBA* expression was reduced to basal levels even in high citrate concentrations, implying that the two-component system is enough to maintain a basal level expression, but is not enough to enhance expression in the presence of citrate. Together, those data showed that citrate-bound BctC was required for both transport and signaling, interacting either with BctE or BctA; a model confirmed in the same study by bacterial two-hybrid assays, showing unprecedented evidence that TctC homologs can be involved also in regulatory processes (Antoine et al., 2005). The presence of citrate responsive regulatory genes and two-component systems adjacent to *tctCBA* operons is not uncommon, as shown for *S. typhimurium* by Widenhorn et al. (1989), for *Comamonas* sp. by Hosaka et al. (2013), for *A. mimigardefordensis* by Wübbeler et al. (2014); and in the genomic searches provided by Antoine et al. (2003). Brocker et al. (2009) also characterized a citrate-responsive two-component system controlling *tctCBA* expression in *C. glutamicum*, although in this case the regulatory proteins were adjacent to another transport system. Interestingly, some of the TTT systems are found in the genome with the *tctB* subunit downstream of *tctA*, such as the *slcHFG* systems from *Roseovarius nubinhibens* (Denger et al., 2009) and *Chromohalobacter salexigens* (Figure 2B, Denger and Cook, 2010). This feature is also observed in some TRAP systems, and systems with this genomic organization are thought to be more similar among them (Mulligan et al., 2011).

The study of two abundantly expressed Bug proteins led to the first crystal structures for TTT family SBPs. These proteins, discussed in detail in the next sections, were fortuitously crystalized with substrates in their binding pocket. BugD contained an aspartate molecule (Huvent et al., 2006a) and BugE contained a glutamate molecule (Huvent et al., 2006b). Amino-acids are the most important carbon and nitrogen sources for *B. pertussis*, which is incapable of metabolism of substrates through the glycolytic pathway (Huvent et al., 2006b); As the Bug proteins are highly expressed, they might play a crucial role in uptake of core metabolic pathways. Herrou et al. (2007) characterized one of the Bug proteins (Bug27), found to be overexpressed in the presence of nicotinic acid, an essential vitamin and a negative modulator of *B. pertussis* virulence. It was shown that this protein binds, with an affinity lower than 1 μM, not only to nicotinate, but also nicotinamide, citrate, benzoate and quinaldic acid. This protein generated also the first TTT SBP crystal structure in an unliganded conformation (Herrou et al., 2007). The binding of Bug27 to nicotinic acid/nicotinamide might suggest it plays a role in virulence modulation, either by interacting with a membrane signal protein or simply transporting nicotinic acid to the cytoplasm. Interestingly, Brickman et al. (2017) suggested that another Bug protein, Bug69, might also be related to the uptake of nicotinic acid and related compounds.

Although not the focus of this review, the potential of the TTT family as a new source for biotechnology relevant uptake systems was also exposed by the genomic analysis performed by Antoine et al. (2003), where it was observed that in many organisms, the *bug* homologs were located near operons that conferred specific abilities to each strain, such as catechol degradation, showing that the importance of this family is wider than the suggested so far and that its diversity might correlate with the metabolic versatility and adaptability of an organism. In addition, a genomic search regarding arylmalonate decarboxylases (AMDases) by Maimanakos et al. (2016) found several members of the TTT family in the vicinity of these enzymes for five of the eight predicted AMDase clusters, either as orphan proteins, in the case of β-proteobacteria; or complete systems, in the case of α-proteobacteria, suggesting the TTT proteins might act to import the carboxylated substrates for subsequent catalysis by the AMDases. Other biotechnologically relevant discoveries include sulfolactate metabolic pathways in *R. nubinhibens* (Denger et al., 2009) and *Chromohalobacter salexigens*, which contain a TTT uptake system for this substrate (Denger and Cook, 2010) named *slcHFG*; a TTT system from *Comamonas* sp., TpiBA and TphC, able to uptake terephthalate (Hosaka et al., 2013); the TctCBA from *A. mimigardefordensis* able to uptake the synthetic molecule disulfide 3,3′-dithiodipropionic acid (DTDP), a precursor for synthetic polythioesters (Figure 2B, Wübbeler et al., 2014); a *tctA* homolog genetically proximal to genes coding for esterase enzymes that degrade organophosphates and potentially related to aromatic compound degradation (Batista-García et al., 2014); the TctABC system from *Halomonas* involved in galactarate/glucarate metabolism (Leyn et al., 2017); and a recent discovery from our group of AdpC, an “orphan” SBP from *R. palustris* which binds medium chain-length dicarboxylic acids ranging from adipate (C6) to azelate (C9) (Rosa et al., 2017). Searching the Enzyme Function initiative (EFI) database, it was observed that 19 homologs of TctC were in their library, however only one of them, a TctC homolog from *Polaromonas* sp. was crystalized in the open apo conformation (PDB accession code 4X9T). Table 1 summarizes the known range of characterized TTT systems with their respective ligands. Initially believed to bind exclusively to citrate, the substrate range for the TTT family is clearly much broader and new substrates are continually being found. With the exception of nicotinic acid in Bug27 (Herrou et al., 2007), all substrates characterized so far seem to have two carboxylic groups, or other functional groups such as sulfate and amide, and further studies will show if this is indeed a required property for substrates in the TTT family.

**Table 1:** Experimentally characterized TTT transporters and SBPs.

**Name(s)**	**Organism(s)**	**Ligand**		**PDB code**	**Reference**
TctCBA	*Salmonella typhimurium*	Citrate	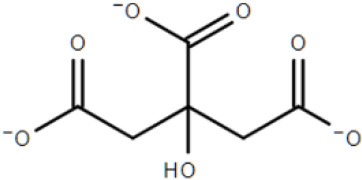		Sweet et al., 1979
BctCBA	*Bordetella pertussis*				Antoine et al., 2003
TctCBA	*Corynebacterium glutamicum*				Brocker et al., 2009
TctCBA	*Commamonas* sp.				Hosaka et al., 2013
TctCBA	*Geobacillus thermodenitrificans*				Graf et al., 2016
BugD	*Bordetella pertussis*	Aspartate	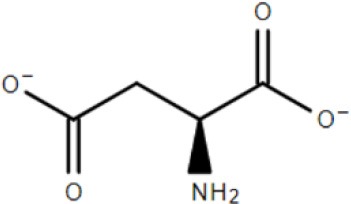	2F5X	Huvent et al., 2006a
BugE	*Bordetella pertussis*	Glutamate	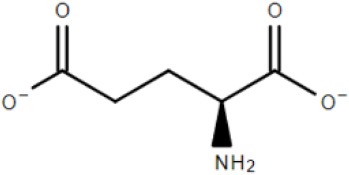	2DVZ	Huvent et al., 2006b
Bug27	*Bordetella pertussis*	Nicotinic acid et al.	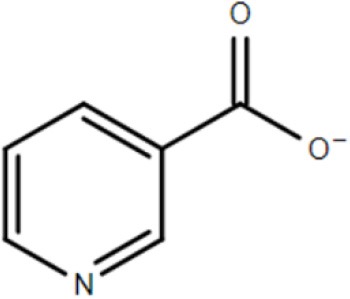	2QPQ	Herrou et al., 2007
SlcHFG	*Roseovarius nubinhibens*	Sulfolactate	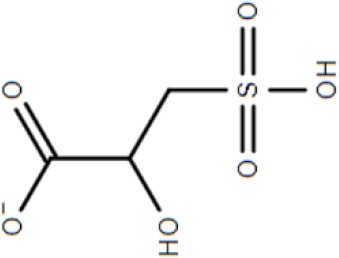		Denger et al., 2009
SlcHFG	*Chromohalobacter salexigens*				Denger and Cook, 2010
TpiBa/ TphC	*Commamonas* sp.	Terephtalate	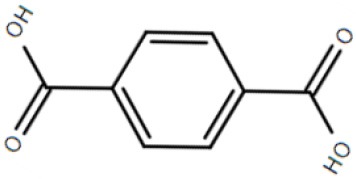		Hosaka et al., 2013
TctCBA	*Advenella mimigardefordensis*	disulfide 3,3′-dithiodipropionic acid (DTDP)	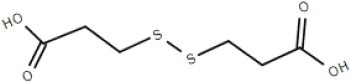		Wübbeler et al., 2014
TctC	*Polaromonas* sp.	Unknown		4X9T	
TctABC	*Halomonas* sp.	Galactarate/glucarate	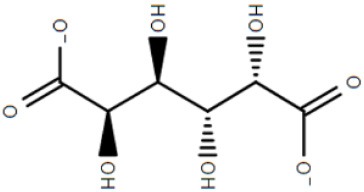		Leyn et al., 2017
AdpC	*Rhodopseudomonas palustris*	Adipate et al.	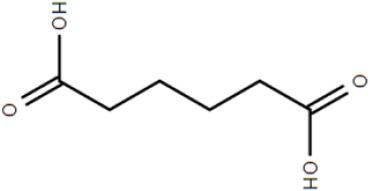	5OEI	Rosa et al., 2017

### Properties and function of the TctAB subunits

The TTT systems are predicted to contain two membrane proteins, homologous to TctA and TctB. Although crystal structures of these proteins have not been elucidated, information from the primary and secondary sequences of these subunits were studied, in addition to some physiological characterizations, in an attempt to understand the energetic and structural mechanisms of the TTT family.

Winnen et al. (2003) showed that while TctB and TctC showed only 27 and 31% identity on average between family members, respectively, TctA orthologs suggested 42% identity and 53% similarity in similar comparisons. Topology predictions suggested the number of transmembrane helices in TctA homologs might vary in different systems, ranging from 9 to 12 in bacteria, and 7 to 11 in archaea. In either group, the N-terminal side was predicted to be in the cytoplasm, and large hydrophilic loops between helices 2 and 3 are conserved among all organisms analyzed, suggesting this region must have an important role in protein function. The motif G-Hy_3_-^*^G-Hy_3_-^*^G-Hy_2_-^*^P-G-Hy-G, where Hy is an aliphatic hydrophobic residue and ^*^ means a fully conserved residue, are found to be highly conserved both in TM1 and TM7, suggesting the 12-TM protein originated from a duplication in a 6-TM ancestor. TctB homologs were predicted to have between 4 and 5 transmembrane domains, also with predicted cytoplasmic N-termini, and are very poorly conserved among bacteria and were not observed in archaeal sequences (Winnen et al., 2003). In a systems biology study comparing a wild type and a multi-drug resistant strain of *Salmonella enterica*, Ricci et al. (2012) observed a G109S SNP in TctA. Although shown to be unrelated to the antimicrobial resistance, this mutation compromised growth on several carbon sources, and curiously seemed to confer a delay in the production of Reactive Oxygen Species (ROS) under stressful conditions. Our genome searches revealed for the first time, that in at least one case, in *Paraburkholderia caribensis*, a fusion between the two membrane subunits can also be observed, forming a single TctAB protein (Figure 2B), similar to what was described for some DctQM proteins from the TRAP family, in particular the ones constituting TAXI-TRAP systems (Mulligan et al., 2011).

Evidence of cation dependence for transport in the TTT family was first provided by Sweet et al. (1979), showing that binding of citrate to TctC was enhanced in the presence of Na^+^ Ca^2+^, Mn^2+^, and Fe^2+^, while Mg^2+^, Ni^2+^, Zn^2+^, and Co^2+^ inhibited uptake. Similarly, Brocker et al. (2009) demonstrated that citrate transport by a TctCBA system of *C. glutamicum* was enhanced by Ca^2+^ and Mg^2+^, but not Sr^2+^. Later studies by Hosaka et al. (2013), however, showed that both the addition of protonophores and an alkaline pH disrupted terephthalate uptake by the TpiBA system in *Comamonas* sp., while deletion experiments in the same study showed that both subunits were essential for substrate uptake. Furthermore, terephthalate uptake was not disrupted when *Comamonas* sp. was grown in absence of Na^+^, suggesting that at least this particular process was more dependent on the proton-motive force rather than a sodium gradient, distinct from SiaPQM sialic acid TRAP transporters (Mulligan et al., 2009).

Discovering a *tctA* homolog next to esterase genes in metagenomics searches, named *tctA_ar*, Batista-García et al. (2014) attempted to build a structural model for TctA_ar based on its primary sequence, given that this protein showed no considerable homology to any other secondary transporter available in the database. The TctA homolog of *Comamonas* sp., characterized in previous studies (Hosaka et al., 2013), was used as a control and called TctA_ct. Two templates were used against each sequence (PDB codes 3VVN and 4K1C), resulting in 4 models in total. In addition to the already described duplicated motif, a high degree of identity was observed between residues 60 and 110 in TctA homologs. The computational models agreed with the predicted 12-TM domain protein, and it was proposed in the 3VVN-based models that the 20 residue conserved motif were in the vicinity of the predicted binding pocket of the protein. In addition, G106, mutated in previous studies (Ricci et al., 2012), would be located between the two repeats, being involved in the beginning of the translocation pathway. In the 4K1C model, the two copies of the domain would be in contact with each other in a helix, which would facilitate conformational changes during the transport cycle. Moreover, given that the TTT transporters were initially known for the transport of citrate, docking of this molecule into the two models was attempted, in addition to the modeling of a Na^+^ binding pocket, suggested to be necessary for citrate transport (Sweet et al., 1979). It was shown in the 3VVN models that the potential Na^+^ binding pockets were located in C-terminal variable regions, while the citrate-binding pocket differs in each of the two proteins. The TctA_ar showed a unique pocket for citrate, while TctA-ct showed several along the predicted channel, which might act as different steps in the translocation pathway. In the 4K1C models, several binding pockets were predicted for citrate in both proteins, with at least one positively charged residue to interact with the ligand in each of them. These models are an important step toward understanding of translocation mechanisms in the TTT family, although biological confirmation of the transport mechanisms in this family are clearly essential.

Evolutionary studies on the TTT family performed by Winnen et al. (2003) suggested that the TctA protein is the original core transport protein, and that the TctB homolog worked as an accessory protein. This claim is supported by the presence of TctA homologs in Archaea, added to the absence of TctB or TctC homologs, and reinforced by the model studies performed by Batista-García et al. (2014). However, the findings on the TRAP transporters, where the DctQ subunit is also observed in archaea, and was found essential to function challenge this hypothesis (Mulligan et al., 2011, 2012). Because the archaea harboring TctA homologs were found in extreme environments, amongst groups of methanogens, hyperthermophiles, and halophiles, it was suggested that the TctA homologs would be involved in a range of different specific metabolic niches (Winnen et al., 2003), but experimental evidence is still lacking to show if this is the case.

### Crystal structure and substrate coordination in TctC homologs

The binding proteins from the TTT family, homologous to TctC (Sweet et al., 1979), show a conserved size (ranging from 29 to 33 kDa), topology and secondary structure organization, but differ considerably in the primary sequence, where among them an identity around 30% is observed. Consequently, a big difference in overall pI is also seen, ranging from 5 to 9.6 (Antoine et al., 2000). At the time of writing, only six structures of TTT SBPs have been deposited in the Protein Data Bank (PDB), four of them with a substrate in the binding pocket; it is already possible to identify, however, some common features among them. The average of 300 amino-acid residues comprises the mature form of the proteins (without signal peptide), separated into two globular domains. Domain one is usually formed by residues 1~100 and 230~300 from the N and C termini, forming a β-sheet of five strands, with topological arrangement β2-β1-β3-β9-β4, surrounded by ~6 α-helices. Domain 2 is comprised of residues 100~229, forming also a central β-sheet of 5 strands with topological arrangement β6-β5-β7-β4-β8, surrounded by ~4 helices. Domain 2 sometimes contains a disulphide bridge between cysteine residues located in α5 and β7, but this is not a feature common to all proteins (Huvent et al., 2006a; Rosa et al., 2017). The junction of the two domains is formed by two β-strands, S4 and S9, which are part of domain 1 but extend up to domain 2, and hydrogen bonds between the two domains are scarce. All these features show that TTT SBP characterized so far can be classified into the Type II binding protein group, according to the scheme of Fukami-Kobayashi et al. (1999), or cluster E-II, accordingly to the new division proposed by Scheepers et al. (2016). Figure 3B shows the crystal structure of BugD as a representative for the TctC homologs. Upon binding to the substrate, it was estimated that the two domains close in an angle of 24.7°, based on the structure of the unliganded nicotinic acid binding protein Bug27 (Herrou et al., 2007). Although TctC proteins most commonly seem to bind to molecules containing carboxylic groups, curiously there is usually a slight overall negative charge in the binding pocket, likely dissipated by the water molecules or dipole effects of the surrounding helices (Rosa et al., 2017). Two β-turns, between β1 and α1; and β7 and α7, form a “pincer-like” structure important in substrate coordination, closing around one carboxylic group of the ligand, while the remainder of it is buried in the pocket (Figure 3). The residues present in the loops characterize distinguishing signatures for proteins of this family, with the motif [P^*^-F-X-A-G^*^-G^*^-X-X-D^*^] in domain 1 being almost ubiquitous among the protein sequences, where X means any residue and ^*^ means a very conserved residue. The backbone atoms of residues in this region seem to make hydrogen bonds with two water molecules, present in all substrate-containing structures and also very well-conserved in position. These water molecules bridge hydrogen bonds between the protein main chain and the proximal carboxylic group in the substrate. This pattern is observed in the coordination of adipate by AdpC (Rosa et al., 2017), aspartate in BugD (Huvent et al., 2006a) and glutamate in BugE (Huvent et al., 2006b). In some Bug protein sequences, although these residues are not conserved, they are substituted by others where the side-chain would contain a hydroxyl group, potentially maintaining hydrogen bonds in similar position to what would be expected of the water molecules (Huvent et al., 2006a). As shown by Herrou et al. (2007), the two β-loops which form the “pincer-like” structure and the two water molecules are not well defined when the ligand is not present. The coordination of the ligand's distal carboxylate group, buried in the pocket, is much less conserved, with α3 and α5 helices apparently varying in position to accommodate each substrate (Huvent et al., 2006a; Rosa et al., 2017). Although not conserved in sequence or topology, the involvement of water molecules in the coordination of the distal carboxylate groups was observed in all cases. In some proteins, hydroxyl groups from threonine and serine residues also form hydrogen bonds with the carboxylate in the substrate, but their positions vary. The carbon chain of the substrate, on the other hand, is stabilized by much more conserved hydrophobic interactions, such as Phe14 in AdpC, which seem to act as a docking site for the substrate, and two glycines (Gly18 and Gly163 in AdpC). As a dynamic model, Herrou et al. (2007) suggested that the unliganded form of the SBP would be in an open conformation, with the “pincer-like” structures flexible. Substrate would then bind to domain 1, which would cause a conformational change that would bring the water molecules and domain 2 together. A comparison between the five available TctC homologs available in the PDB (only one AdpC structure was used) is shown in Table 2. The root mean square deviations (RMSD), as expected, show a bigger difference when comparing closed and Apo structures, and smaller RMSDs when comparisons were made between two liganded structures. Taken together, the crystal structures presented to date give a good general mechanism for ligand coordination in the TctC homologs, and further studies will enable us to validate this model and detail the potential differences for substrates containing different functional groups, such as nicotinamide (Herrou et al., 2007) and sulfolactate (Denger and Cook, 2010).

**Table 2:** Comparison between the TctC homologs structures available in the PDB.

**RMSDs (Å)**	**BugD (B)**	**BugE**	**Bug27 (B)**	**TctC**	**AdpC**
**SI (%)**	**2F5X**	**2DVZ**	**2QPQ**	**4X9T**	**5OEI**
BugD (B)	NA	1.33	3.00	3.89	1.75
BugE	34	NA	2.62	3.56	1.54
Bug27 (B)	25	32	NA	2.2	2.75
TctC	15	19	28	NA	3.96
AdpC	30	33	27	18	NA

*Root Mean Square Deviations (RMSD) and Sequence Identity (SI) were generated using EMBL 3D alignment tool available at http://www.ebi.ac.uk/msd-srv/ssm/cgi-bin/ssmserver. PDB codes for each of the structures is shown below the protein names in the horizontal line*.

### Present in abundance: the overrepresentation of TctC homologs in some bacteria

The limited number of sequences available at the time resulted in a bias in the studies performed by Winnen et al. (2003), which suggested that the TTT systems were mostly present in α-proteobacteria, and that most other bacterial groups had few or no homologs of these proteins. Genomic searches following the discovery of BugT by Antoine et al. (2000), however, revealed the *bug* genes to be very overrepresented in *B. pertussis*, with 79 BugT homologs, making this family the most abundant in the genome. Following this discovery, Antoine et al. (2003) performed a wider genome analysis, showing that this overrepresentation was extended to several *Bordetella* species, and that some of the Bug proteins were also among the most abundant in cell protein extracts in *B. pertussis*. As stated in previous sections, the numbers of TTT transmembrane components did not follow the same process, being found in small numbers and consequently configuring most BugT homologs as “orphan proteins,” with no obvious transmembrane counterparts. The existence of orphan *bug* homologs was also observed by Antoine et al. (2003) in the genomes of several other bacteria genera, although in that study the only *bacterium* shown to have as many representatives as the *Bordetella* species was the β-proteobacterial relative *Cupriavidus metallidurans*. At the time that search was performed, there were around 200 complete bacterial genomes available in the databases, and more recent genome releases showed that at least two other β-proteobacteria genera, *Advenella* and *Cupriavidus* also showed an overrepresentation of *tctC* homologs (Wübbeler et al., 2014). For this review, we reassessed the distribution of TTT systems using the 8049 fully assembled genomes in Genbank, of 2,323 different species, to provide an updated analysis of the presence of TTT systems in bacterial genomes.

A total of 2,323 complete bacterial genomes retrieved from the NCBI database, one per species, were screened for TctA and TctC homologs using the TBLASTN tool. Searches were performed against the coding sequence (CDS) database of each species using lists of protein sequences of either TctA or TctC homologs as queries rather than single sequences, in order to avoid query bias. For the TctC search, the queries were: TctC from *S. enterica* (Sweet et al., 1979); BugD, BugE, and Bug27, from *B. pertussis* (Herrou et al., 2007), TphC fom *Comamonas* sp. (Hosaka et al., 2013) and AdpC from *R. palustris* (Rosa et al., 2017). For the TctA search, we used the TctA from *S. enterica* (Sweet et al., 1979), *A. mimigardefordensis* (Wübbeler et al., 2014), and *C. glutamicum* (Brocker et al., 2009); the BctA from *B. pertussis* (Antoine et al., 2005); the TpiA from *Comamonas* sp. (Hosaka et al., 2013) and the SlcF from *R. nubinhibens* (Denger et al., 2009). Due to the poor sequence conservation among the TctB proteins, our searches with this subunit proved to be unsuccessful. TBLASTN reports were obtained for a range of *E*-values from 1 to 10^−15^ in order to determine the best threshold to avoid spurious hits, while still retaining distant paralogs. The complete table with number of hits for both proteins with an *e*-value of 10^−15^ is presented in Supplementary Table 1.

**Figure 4:**
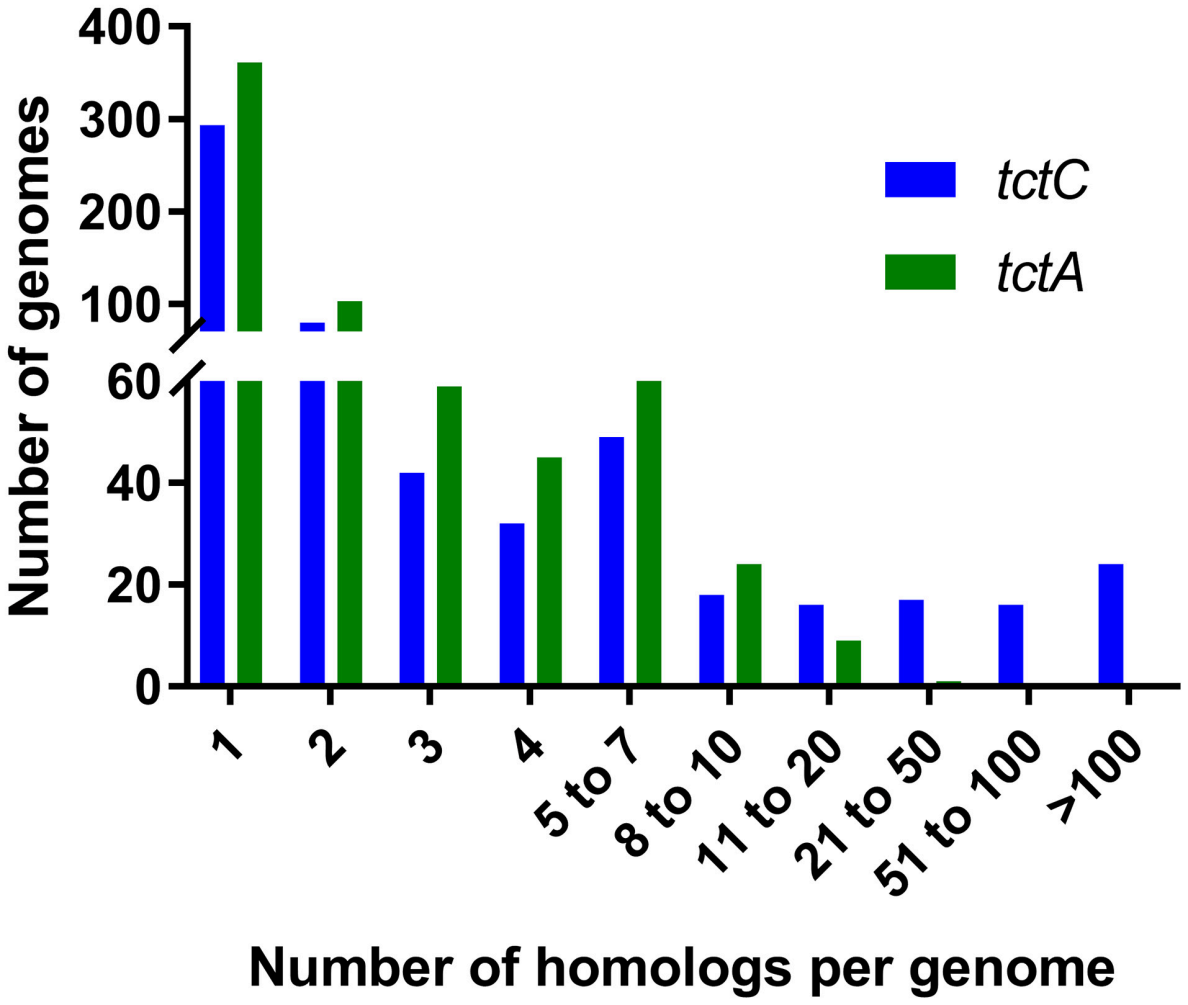
Numbers of genomes containing different ranges of homologs for *tctC* and *tctA*.

Our searches revealed that, in accordance with the findings of Antoine et al. (2003) and further reinforced by Wübbeler et al. (2014), the most extreme examples of overrepresentation of this group of proteins are found among β-proteobacteria, especially among *Bordetella* species, as shown in Table 3. However, this phenomenon is not restricted to this group, but extends also to species in the α-proteobacteria class (Table 3). In fact, the genome of the environmental α-proteobacterium *Rhodoplanes* sp. encodes 434 TctC homologs in its 8.2 Mbp genome, more than double that of some *Bordetella* species. A more detailed investigation of the expansion in *Rhodoplanes* will be reported elsewhere (manuscript in preparation). Although this analysis shows that the overrepresentation of TctC homologs is mostly found in proteobacteria, a deeper phylogenetic analysis is still required in order to clarify whether this feature found in different subgroups originates from duplications in a common ancestor or were independent events resulting from convergent evolution and independent multiplication events. A search for TctA homologs, on the other hand, as shown in Table 4, suggests that the genomes containing the largest numbers of homologs are found among α and γ-proteobacteria, with only two β-proteobacteria showing 8 or more homologs. In this search, the top hits are no higher than 21 per genome, and are usually associated with a similar number of TctC homologs, possibly forming complete tripartite systems. In this case, organisms outside of the class of proteobacteria, such as clostridia, spirochaetes, and bacilli are also observed to harbor these homologs. An overview of the number of genomes encoding different numbers of TctC and TctA homologs are shown in Figure 4, and the full complement of genomes analyzed is shown in Supplementary Table 1.

**Table 3:** Number of TctC and TctA homologs per accession.

**Species**	**Class**	***tctC* homologs**	***tctA* homologs**
*Achromobacter denitrificans*	β-proteobacteria	99	6
*Achromobacter insolitus*	β-proteobacteria	177	4
*Achromobacter xylosoxidans*	β-proteobacteria	202	5
*Acidovorax avenae*	β-proteobacteria	66	4
*Acidovorax citrulli*	β-proteobacteria	55	4
*Advenella kashmirensis*	β-proteobacteria	116	1
*Advenella mimigardefordensis*	β-proteobacteria	129	7
*Alicycliphilus denitrificans*	β-proteobacteria	143	3
*Bordetella bronchialis*	β-proteobacteria	195	5
*Bordetella bronchiseptica*	β-proteobacteria	182	4
*Bordetella flabilis*	β-proteobacteria	214	6
*Bordetella genomosp*	β-proteobacteria	139	4
*Bordetella hinzii*	β-proteobacteria	105	5
*Bordetella holmesii*	β-proteobacteria	56	6
*Bordetella parapertussis*	β-proteobacteria	142	4
*Bordetella pertussis*	β-proteobacteria	81	2
*Bordetella petrii*	β-proteobacteria	107	5
*Bordetella pseudohinzii*	β-proteobacteria	86	5
*Bordetella trematum*	β-proteobacteria	100	5
*Bordetella* sp. H567	β-proteobacteria	186	8
*Comamonas serinivorans*	β-proteobacteria	87	1
*Comamonas testosteroni*	β-proteobacteria	100	2
*Cupriavidus basilensis*	β-proteobacteria	155	5
*Cupriavidus gilardii*	β-proteobacteria	98	6
*Cupriavidus metallidurans*	β-proteobacteria	122	5
*Cupriavidus necator*	β-proteobacteria	190	3
*Cupriavidus* sp. USMAHM13	β-proteobacteria	130	4
*Delftia acidovorans*	β-proteobacteria	157	3
*Delftia* sp. CS1_4	β-proteobacteria	152	3
*Delftia tsuruhatensis*	β-proteobacteria	145	3
*Hydrogenophaga* sp. PBC	β-proteobacteria	64	4
*Polaromonas* sp. JS666	β-proteobacteria	96	4
*Pseudorhodoplanes sinuspersici*	α-proteobacteria	99	8
*Pusillimonas* sp. T7-7	β-proteobacteria	52	3
*Ralstonia eutropha*	β-proteobacteria	156	5
*Ramlibacter tataouinensis*	β-proteobacteria	75	3
*Rhodoferax* sp. DCY110	β-proteobacteria	90	3
*Rhodoplanes* sp. Z2	α-proteobacteria	434	9
*Variovorax paradoxus*	β-proteobacteria	135	8
*Verminephrobacter eiseniae*	β-proteobacteria	130	3

*Only accessions with more than 50 TctC homologs are presented. An e-value of 10^−15^ was used*.

**Table 4:** Number of TctC and TctA homolog per accession.

**Species**	**Class**	***tctA* homologs**	***tctC* homologs**
*Antarctobacter heliothermus*	α-proteobacteria	12	9
*Bordetella* sp. H567	β-proteobacteria	186	8
*Bradyrhizobium icense*	α-proteobacteria	8	43
*Chelativorans* sp. BNC1	α-proteobacteria	13	15
*Chelatococcus* sp. CO-6	α-proteobacteria	11	10
*Chromohalobacter salexigens*	γ-proteobacteria	8	9
*Defluviimonas alba*	α-proteobacteria	9	9
*Desulfovibrio fairfieldensis*	δ-proteobacteria	9	9
*Ensifer sojae*	α-proteobacteria	9	6
*Geosporobacter ferrireducens*	Clostridia	8	9
*Granulosicoccus antarcticus*	γ-proteobacteria	13	13
*Halomonas chromatireducens*	γ-proteobacteria	8	8
*Halomonas huangheensis*	γ-proteobacteria	17	14
*Kushneria konosiri*	γ-proteobacteria	9	9
*Kushneria marisflavi*	γ-proteobacteria	9	9
*Lachnoclostridium* sp. YL32	Clostridia	8	5
*Marinobacterium aestuarii*	γ-proteobacteria	13	11
*Marinomonas* sp. MWYL1	γ-proteobacteria	9	7
*Marinovum algicola*	α-proteobacteria	14	12
*Martelella mediterranea*	α-proteobacteria	21	18
*Martelella* sp. AD-3	α-proteobacteria	10	9
*Oligotropha carboxidovorans*	α-proteobacteria	8	8
*Paenibacillus naphthalenovorans*	Bacilli	16	19
*Pelagibacterium halotolerans*	α-proteobacteria	8	7
*Pseudorhodoplanes sinuspersici*	α-proteobacteria	8	99
*Rhodoplanes* sp. Z2	α-proteobacteria	9	434
*Sediminispirochaeta smaragdinae*	Spirochaetes	9	9
*Sinorhizobium fredii*	α-proteobacteria	8	7
*Sphaerochaeta globosa*	Spirochaetes	9	6
*Sphaerochaeta pleomorpha*	Spirochaetes	9	7
*Starkeya novella*	α-proteobacteria	15	17
*Sulfitobacter pseudonitzschiae*	α-proteobacteria	8	7
*Variovorax paradoxus*	β-proteobacteria	8	135
*Yangia* sp. CCB-MM3	α-proteobacteria	8	6

*Only accessions with 8 or more TctA homologs are presented. An e-value of 10^−15^ was used*.

At an *e*-value of 10^−15^ in BLAST searches, the TctC homologs outnumber the TctA homologs in 176 genomes, as shown in Table 5. As already discussed, in these cases it might be that one TctA interacts with more than one TctC, or that the latter are involved in processes other than transport, such as signaling and chemotaxis (Antoine et al., 2003; Piepenbreier et al., 2017). These latter suggestions are reinforced by the fact that in 36 genomes, one *tctC* homolog was found, but no *tctA* homologs, although the hypothesis that the binding proteins might interact with transmembrane domains of other transporter classes cannot be excluded. In our initial searches, we found that 210 genomes showed an excess of *tctA* homologs in relation to *tctC*, an unprecedented observation to the best of our knowledge. In order to see whether these observations were due to too strict threshold, our searches were repeated with different *e*-values, shown in Table 5. As shown, using an *e*-value of 10^−9^, the number of genomes where this situation occurs is reduced to 66, and to 39 in 10^−6^. At the latter threshold, 6 genomes indicated the presence of TctA homologs, but no TctC homologs. Investigating these 6 genomes individually, we found that 4 of them contained a truncated *tctC* homolog in the vicinity of the *tctA* gene and in one the *tctA* gene was clearly mutated. The single remaining genome, *Mageeibacillus indolicus*, indeed seems to have no indication of any periplasmic binding proteins in the vicinity of the *tctA* gene. If the existence of a *tctA* gene without any *tctC* is not a search artifact, one possible explanation would be that SBPs of other types of transport systems could be capable of interacting with the TTT transmembrane subunits. An alternative is that such rare orphan TctA proteins in bacteria are, like in archaea, capable of functioning without the involvement of an SBP (Winnen et al., 2003), although both the TTT systems characterized so far and the experiments with the TRAP transporters suggest otherwise (Brocker et al., 2009; Mulligan et al., 2009; Hosaka et al., 2013). In another 316 genomes, the numbers of *tctC* and *tctA* genes match exactly, suggesting all proteins would be involved in transport through tripartite systems. Finally, about half of the genomes searched (1621) contained no homologous proteins to any of the queries used, suggesting, given TctA homologs are also found in archaea (Winnen et al., 2003), that TTT systems were lost during evolution in these phylogenetic branches.

**Table 5:** Number of genomes showing different patterns in terms of numbers of *tctC* and *tctA* homologs, using different *e*-values as thresholds.

***e*-value**	**10^−15^**	**10^−12^**	**10^−9^**	**10^−6^**	**10^−3^**	**10^0^**
*tctC* > *tctA*^a1^	176	196	212	220	255	2,157
*tctA* > *tctC*^b1^	210	100	66	47	39	64
*tctC* without *tctA*^a2^	36	43	54	56	79	338
*tctA* without *tctC*^b2^	115	33	13	6	8	9
*tctC* = *tctA* ≠ 0	316	415	444	457	456	97
No homologs	1,620	1,612	1,601	1,599	1,573	5
Total Number of *tctA* homologs	1,633	1,637	1,639	1,641	1,649	5,705
Total number of *tctC* homologs	7,213	7,405	7,495	7,552	7,632	25,225

*^a2^ is also counted inside of ^a1^, and ^b2^ is also counted inside ^b1^*.

The reason for the overrepresentation of the Bug proteins in *Bordetella* species and other Proteobacteria remains unclear. Antoine et al. (2003) suggested that the few transmembrane domains of TTT systems evolved to be poorly specific, being able to interact with several TctC homologs and thus be required for the uptake of different substrates. This hypothesis was also suggested by Hosaka et al. (2013), but no evidence for this mechanism is yet available. One could hypothesize that perhaps many of these proteins have similar binding functions, but are expressed differentially during the infection cycle in *Bordetella* species in order to evade the immune system more efficiently. However, the fact that many environmental Proteobacteria also have this expansion of Bug proteins suggests instead that it is an earlier evolutionary trait. The genome of *Ralstonia eutropha* containing 154 homologs of *tctC*, reveals that the majority of them (64.1%) have in their vicinity a regulatory protein, suggesting that most of these proteins are associated with regulatory mechanisms rather than transport (Pohlmann et al., 2006). In this sense, the nomenclature of these SBPs as “uptake genes” might not reflect their actual role in the cell. Piepenbreier et al. (2017) provides a good review of how transporters from different classes can act as first agents in signaling pathways, and further studies will enrich our understanding to whether this is the case for the TTT family.

## Concluding remarks

In this review, we showed how transport systems from the TRAP and TTT families can play important roles in bacteria with a focus on pathogenicity and colonization. Recent high-throughput studies increased substantially the range of substrates known for the TRAP family, while the TTT family is still understudied with a more limited known substrate range, being unraveled in individual studies. In addition, while a lot has been elucidated regarding the binding mechanisms, energetics, and kinetics of the TRAP family, very few equivalent studies exist for the TTT family, where especially the energy-coupling mechanisms are yet to be elucidated properly. For both families, a crystal structure of the complete tripartite systems would greatly increase our understanding of the transport process across the membrane, perhaps with potential applications as new drug targets in pathogenic bacteria, given the absence of these transporters in eukaryotic cells.

## Author contributions

LR reviewed the literature, co-wrote the manuscript and co-analyzed the bioinformatic data. MB generated the bioinformatics data and co-analyzed it. GT edited and commented on the drafts. DK conceived the idea and focus of the review, co-wrote and edited the paper, and provided supervisory guidance.

### Conflict of interest statement

The authors declare that the research was conducted in the absence of any commercial or financial relationships that could be construed as a potential conflict of interest.

## Supplementary material

Supplementary Table 1 Number of genes homologous to *tctC* and *tctA* in 2,323 bacterial genomes, obtained using TBLASTN.

## Abbreviations

SBP: Solute Binding-Proteins
TTT: Tripartite Tricarboxylate Transporters
TRAP: Tripartite ATP-independent periplasmic transporter.
